# Optimum Efficiency of a Steam Ejector for Fire Suppression Based on the Variable Mixing Section Diameter

**DOI:** 10.3390/e24111625

**Published:** 2022-11-09

**Authors:** Yu Han, Xiaodong Wang, Ao Li, Anas F. A. Elbarghthi, Chuang Wen

**Affiliations:** 1 School of Mechanical and Electrical Engineering, Suqian University, Suqian 223800, China; 2 School of Mechanical Engineering & Automation, Northeastern University, Shenyang 110819, China; 3 School of Mechanical and Manufacturing Engineering, University of New South Wales, Sydney, NSW 2052, Australia; 4 Faculty of Environment, Science and Economy, University of Exeter, Exeter EX4 4QF, UK; 5 Faculty of Mechanical Engineering, Technical University of Liberec, Studentská 1402/2, 46117 Liberec, Czech Republic

**Keywords:** fire suppression, steam ejector, diameter, CFD simulation, critical back pressure

## Abstract

The steam ejector is valuable and efficient in the fire suppression field due to its strong fluid-carrying capacity and mixing ability. It utilizes pressurized steam droplets generated at the exit to extinguish the fire quickly and the steam droplet strategy allows for an expressive decrease in water consumption. In this regard, the fire suppression process is influenced by the steam ejector efficiency, the performance of the pressurized steam, and the ejector core geometry, which controls the quality of the extinguishing mechanisms. This study investigated the impact of different mixing section diameters on the pumping performance of the ejector. The results showed that change in the diffuser throat diameter was susceptible to the entrainment ratio, which significantly increased, by 4 mm, by increasing the throat diameter of the diffuser and improved the pumping efficiency. Still, the critical back pressure of the ejector reduced. In addition, the diameter effect was studied and analyzed to evaluate the ejector performance under different operating parameters. The results revealed a rise in the entrainment ratio, then it diminished with increasing primary fluid pressure. The highest entrainment ratio recorded was 0.5 when the pressure reached 0.36 MPa at the critical range of back pressure, where the entrainment ratio remained constant until a certain back pressure value. Exceeding the critical pressure by increasing the back pressure to 7000 Pa permitted the entrainment ratio to reduce to zero. An optimum constant diameter maximized the ejector pumping efficiency under certain operating parameters. In actual design and production, it is necessary to consider both the exhaust efficiency and the ultimate exhaust capacity of the ejector.

## 1. Introduction

As water demonstrates ideal thermal characteristics, it has become the most widely used fire suppression agent and is suitable for most types of fire [1]. Moreover, the phase change from liquid water to steam can be applied to sparkling and water mist fire suppression systems. Concurrently, a steam ejector, as a high-momentum spray generator, allows rapid fire suppression, which can disperse suppressants and minimize fire damage [2]. The ejector mainly comprises a primary conversion diversion nozzle, a mixing chamber with a constant area section, and a diffuser [3]. The geometry and the operating parameters play the leading role in determining the ejector efficiency. However, the properties of the working fluid and the flow pattern structure inside the ejector require a more detailed understanding to improve the ejector pumping efficiency [4]. Most of the available research in this area was achieved through experimental work and numerical simulations. So far, with the continuous development of experimental techniques and methods, the related experiments of the jet refrigeration system have made progress in some aspects [5,6]. It is widely used in the hydrogen energy storage cycle, biopharmaceutical, chemical production and other fields [7]. It can also be used in fire extinguishing and suppression systems to replace fine water mist, providing greater oxygen displacement by the water droplet evaporation from the fire plume [8]. These days, more and more studies, including experimental studies, have been conducted on the pressure, spray rate, cone angle, droplet size and other system characteristics at various working operational modes, based on the nozzle and water mist [9]. Besides numerical studies, compared with the traditional sprinkler, the strategy of using the steam greatly supports saving the amount of water used, reducing the pipe flow quantity and the cost of treatment. Nevertheless, the main significant factor controlling the performance of high-pressure steam, and influencing the fire extinguishing mechanism, is the steam injector nozzle efficiency [10,11,12]. At the same time, improving its work efficiency has become the focus of current research. The water steam jet cooling system represents typical equipment for a clean and environmentally protected means of industrial waste heat and waste heat working medium. The flow process in the water vapour ejector is very complex and changeable. The Laval nozzle accelerates the working medium (steam) from stagnation to supersonic speed, and low pressure is generated at the entrance of the mixing chamber. Under the action of pressure difference, the high-pressure gas in the evaporator is pumped and passed to the mixing chamber, where the intense momentum and energy exchange occurs to create high-speed working steam [13]. Finally, the inlet fluids from both streams are mixed in the mixing chamber and equivalent section and then put into the condenser after the diffuser slows down and pressurizes [14]. The flow behaviour of the mixed stream in the ejector is complex at transonic flow, and there are flow characteristics, such as congestion, shock wave, and boundary layer separation [15,16,17]. Recently, numerical simulations of water vapour ejectors have attracted more and more attention and have become the focus of research. It provides an important reference for improving the efficiency of the ejector and enhancing and optimizing the structure and performance of the ejector. However, in the aspect of experimental study, the experimental study of the ejector is insufficient. Han et al. reported the structure of the internal steam ejector flow [18] and shock wave [19] driven rejected combustion engine heat source. The research revealed the potential effect of the internal structure flow on the entrainment ratio (determined as the ratio between the suction and the motive nozzle mass flow rates), mainly the location of the normal shock and the pseudo-shock region. Therefore, the optimization of the pumping efficiency was identified by the pressure range of the critical primary fluid.

Based on a literature survey of material published in recent decades, the research and experimental data about ejector experiments are still very limited. As a result, the applicability of the theoretical model and the correctness of the numerical method is not verified and supported by experimental data, which limits the generalization and application of the numerical simulation results to a certain extent. From our previous research work, the exhaust performance of steam ejectors is not only affected by the operating parameters: working steam parameters, pumped gas parameters and fluid outlet parameters [20,21]. Its geometric parameters are also core features monitoring the performance of ejectors [22,23,24]. For the ejector, the direction and distance of the equal straight section directly affect the injector’s performance because it is not only working steam and pumped gas mixed through the passage. At the same time, under the condition of congestion, its size is a decisive factor affecting the pumping efficiency [25,26,27]. Based on the establishment of a small ejector refrigeration system for laboratory use, the variation of the ejection entrainment ratio of steam ejectors under different diffuser diameters and different operating parameters was studied. It provides a valuable reference for improving the performance of steam ejectors and optimizing the ejector and provides data support for verifying the numerical simulation model.

By reviewing previous research work, the findings and novelty of this work are summarized as follows:

a) Comprehensive numerical simulations using various mixing section diameters were performed to examine the link between geometry and the steam ejector pumping efficiency.
b) The simulation was validated with experimental results to approve the accuracy of the research. In addition, different turbulent models with supported wall functions were considered for optimization prediction certainty.
c) The fluid flow characteristics under different mixing section diameters were analyzed and discussed in detail.
d) The influence of ejector back pressure on ejector efficiency under different mixing section diameters was analyzed, and the critical back pressure under certain conditions was analysed and discussed.
e) The influence of diameter on flow characteristics was simulated to study the optimization of steam ejector performance under certain operating conditions.

## 2. Numerical Algorithms

### 2.1. Governing Equations

In this study, the flow in the ejector is described as an asymmetric compressible steady state restrained by the conservation equation. In contrast, as in the case of variable-density flow, the Navier–Stokes equation is adopted to solve the flow behaviour [28].

Moreover, the viscous dissipation rate could be defined, together with the ideal gas law, through the total energy equation [29]. It should be recognized that steam has unique transport and thermodynamic properties, which remain unchanged within the remit of the analysis and their significant effects were not found during validation. However, the continuity, momentum and energy equations are described in the simulation and stated as follows:

Continuity equation:

(1) $$\frac{\partial \rho }{\partial t}+\frac{\partial }{\partial x_{i}}\left(\rho u_{i}\right)=0$$

Momentum equation:

(2) $$\frac{\partial }{\partial t}\left(\rho u_{i}\right)+\frac{\partial }{\partial x_{j}}\left(\rho u_{i}u_{j}\right)=-\frac{\partial P}{\partial x_{i}}+\frac{\partial \tau_{ij}}{\partial x_{j}}$$

Energy equation:

(3) $$\frac{\partial }{\partial t}\left(\rho E\right)+\frac{\partial }{\partial x_{i}}\left(u_{i}\left(\rho E+P\right)\right)=\vec{\nabla }\cdot \left(\alpha_{\mathrm{eff}}\frac{\partial T}{\partial x_{i}}\right)+\vec{\nabla }\cdot \left(u_{j}\left(\tau_{ij}\right)\right)$$

where

(4) $$\tau_{ij}=\mu_{\mathrm{eff}}\left(\frac{\partial u_{i}}{\partial x_{j}}+\frac{\partial u_{j}}{\partial x_{i}}\right)-\frac{2}{3}\mu_{\mathrm{eff}}\frac{\partial u_{k}}{\partial x_{k}}\delta_{ij}$$

with

(5) $$\rho =\frac{P}{RT}$$

where *E* represents the total energy, *μ*_eff_ is the effective molecular dynamic viscosity, *τ*_*ij*_ describes the stress tensor, and α_eff_ denotes the effective thermal conductivity.

### 2.2. Ejector Geometry and Mesh Sensitive Analysis

The ejector geometry used in this study is described in detail in this section. The simulation considered a two-dimensional axisymmetric ejector, which proved its efficiency in reducing the calculation time and cost. Table 1 illustrates the main geometrical parameters of the tested ejector.

**Figure 1:**
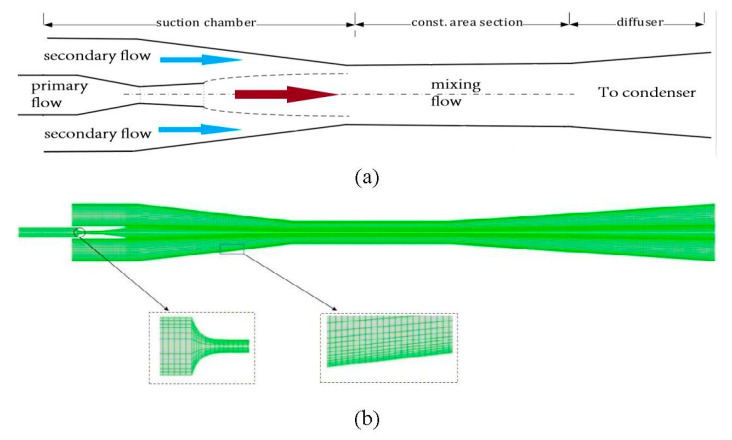
Steam ejector (**a**) schematic view, (**b**) grid structure.

**Figure 2:**
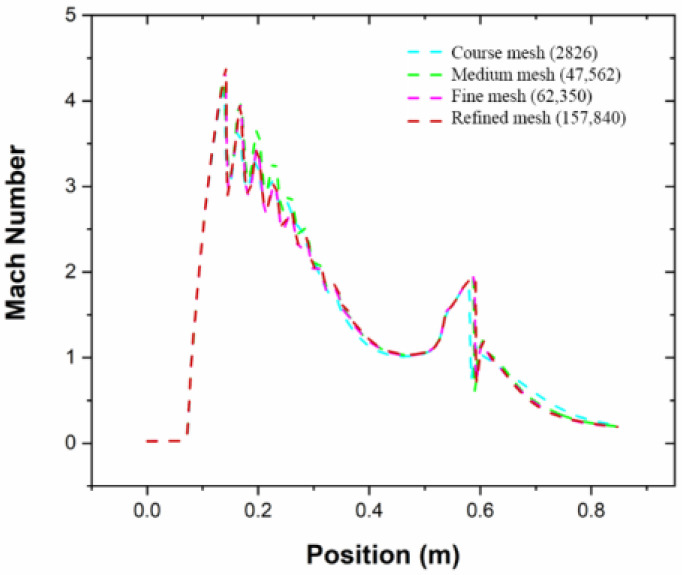
Verification of mesh independence.

The model started with the mesh structure for the ejector flow domain. Intensive effort was given to the location predicted with high local speed to generate high-precision results. Figure 1 emphasizes the adaptive technology to highlight the specific structure of the grid. In this analysis, the grid was created with high quality and with a maximum aspect ratio of 0.9 and 5:1, respectively. These settings ensured an accurate result besides the converging of the solution. Figure 2 illustrates the distribution curve comparison of the Mach number at the centerline using various grid densities. The result revealed a similar trend by applying both the fine and medium grids in the simulations. Therefore, the medium grid model was selected for this study with 47,562 elements because it was independently verified, which contributes to improving computational efficiency.

### 2.3. Numerical Simulation Settings

The commercial Computational Fluid Dynamics (CFD) using ANSYS Fluent was employed as a platform for simulation. The research work by Pianthong et al. reported relatively identical results obtained from both the 3-D and 2-D axisymmetric models [30]. Hence, the 2D axisymmetric model was used in this study to save computational time and gain consistent solutions. In practice, there is spontaneous condensation of steam inside the ejector. Therefore, the ideal gas model was used in this paper to probe for physical insights through simulation analysis.

The flow inside the ejector is described as a compressible steady state governed by energy, mass, and momentum equations. The density-based solver was used because it proved excessive stability in the literature. Since high compressible flow and shock waves would exist, the SST k-ω turbulence model was selected. The boundary conditions were chosen to be pressure-inlet for the steam inlet, pressure-outlet for the exit, and adiabatic no-slip wall boundary conditions supported with Enhanced Wall Treatment. The second-order upwind discretization was applied for all the convective terms over the simulation.

## 3. CFD Model Verification

The geometric parameters of the steam ejector were gained from our previous experimental study, shown in Table 1.

For the validation of the simulation result, the distribution of the static pressure along the ejector wall was used. The pressure values used in the comparison at the primary and the secondary flow streams were set at 0.34 MPa and 1.71 kPa, respectively, via different back pressure ranges.

**Figure 3:**
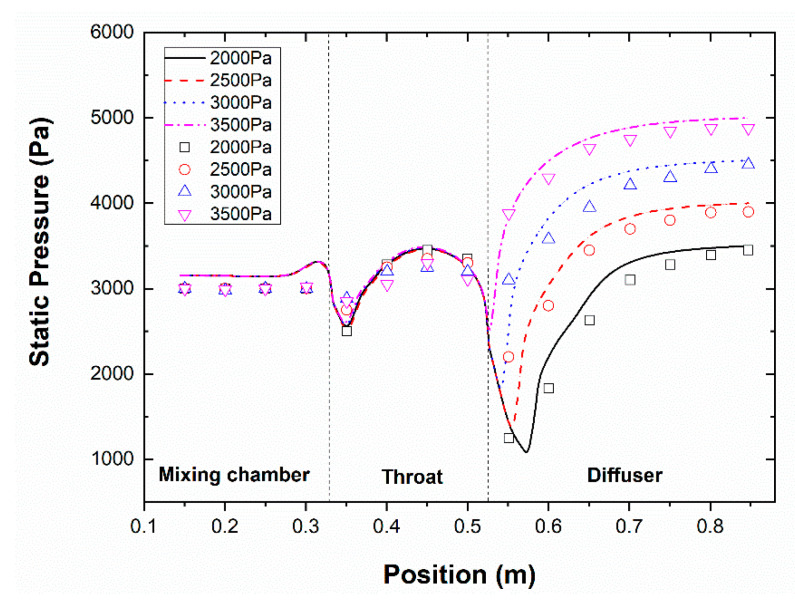
Static pressure distribution at the wall via different back pressure.

It is observable from Figure 3 that the experimental and the simulated distribution of the static pressure results were similar. The CFD result represented a maximum deviation of 8% compared with the experimental data, which supported the analysis of the current research, based on the allowable error in the engineering sector of the study. There was a particular gap between the experimental and the simulated values, and the former was slightly higher than the latter. However, a particular difference takes place in the ejector mixing chamber. The reason could be explained by two points. First of all, the use of the ideal gas model. The other reason refers to the experimental sealing property of the test rig, including the manufacturing error. However, the result obtained from the simulation supported the analysis of this paper in an acceptable range. The region of the divergent and the throat section represented good agreement for the wall pressure distribution with a relatively consistent trend which verified the accuracy of the CFD technique. Generally, the main reasons for the error obtained in the simulation could be explained from the experimental data side; for instance, the installation error, the instrument error, the measurement error, and the error of the steam source. Throughout the test, the temperature of the steam in the evaporator was measured with thermocouples. It should be clarified that the steam temperature changed dynamically close to the set temperature location affected by the ambient and the inlet temperatures measured by the thermocouples.

## 4. Results and Discussion

### 4.1. The Effect on the Boundary Layer Separation

**Figure 4:**
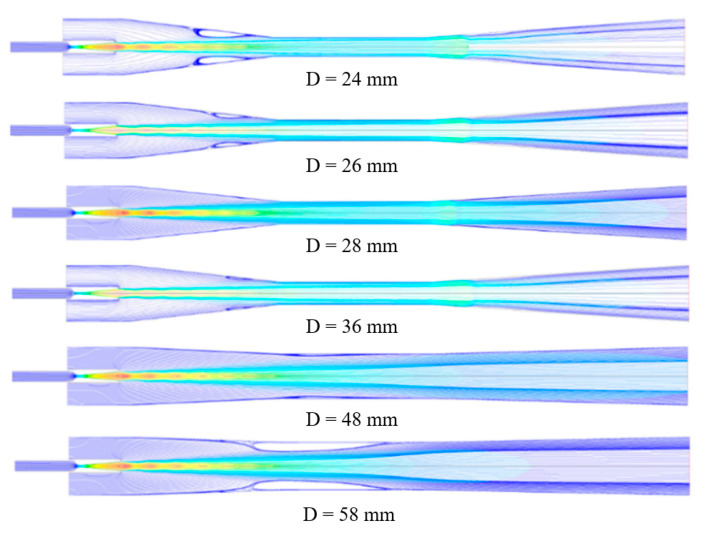
The impact of different mixer diameters on the boundary layer separation.

Figure 4 shows the local Mach number contour of the steam ejector under different mixing section diameters. The boundary conditions for these simulations included the following: (a) the pressure and temperature at the primary inlet were 360 kPa and 135 °C, (b) the pressure and temperature at the secondary inlet were 1710 Pa and 10 °C, and (c) the back pressure at the outlet was 3500 Pa. It can be seen that no boundary layer separation condition occurred in the mixing chamber of the ejector when the diameter varied from 36 mm to 48 mm. The limited flow area of the secondary fluid did not change. It also showed the interaction between the fluid in the ejector and the wall was small in the range of diameter variation. The gradient of the velocity and pressure was small, and the motion between them was relatively stable. When the diameter was less than 36 mm, the diameter size decreased. The mixing chamber was the only location where the boundary layer separation occurred, and the separation degree increased gradually with the diameter decrease. The main reason was that as the diameter decreased, the friction between the ejector wall and the mixed fluid increased, and the velocity difference became larger. The velocity difference resulted in vortices between the secondary fluid and the wall. With the continuous expansion of the vortex, the boundary layer separation degree gradually rose.

**Figure 5:**
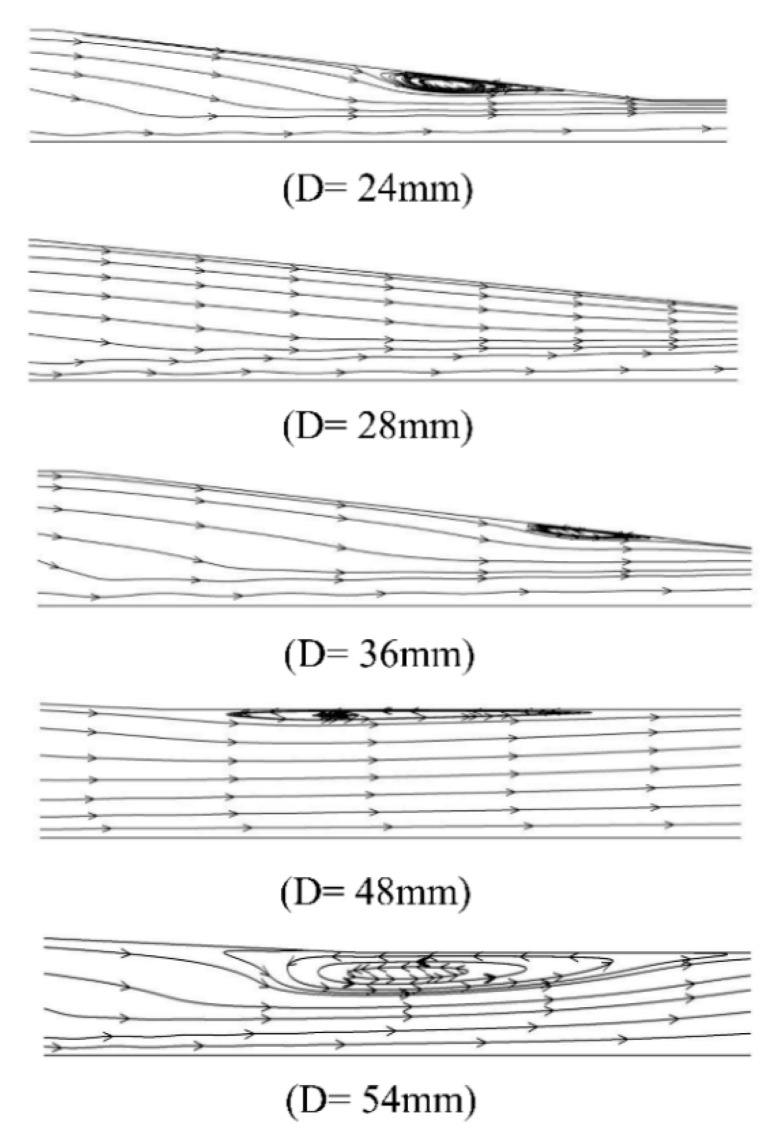
The vortex phenomenon at different mixer diameters.

Moreover, the boundary layer separation degree witnessed a decrease when the diameter became larger than 48 mm. It also extended to the mixed section. Figure 5 indicates the magnified trace of boundary layer separation. The result demonstrated that the growth of the diameter values enhanced the vortex generated by the inverse pressure gradient, and consequently, the boundary layer separation was aggravated.

The reason was that with the increase of the diameter, the flow channel of the fluid became wider. The ejection steam energy near the wall was small, and the force between the walls increased. Then, a large pressure gradient was generated between the primary stream and the wall surface. Accordingly, pressure vortices were generated near the wall surface, leading to the separation of the boundary layer.

The occurrence of boundary layer separation led to the compression of the flow passage of the secondary fluid. The mass flow rate of the primary fluid remained unchanged under the condition of fixed inlet primary stream conditions. Therefore, the entrainment ratio was reduced, and the ejector pumping efficiency decreased. As can be seen from Figure 5, when the mixing section diameter was less than 36 mm, the boundary layer separation existed in the “separation zone”. It is described as the area from the separation point in the mixing section to the reattachment point. However, once the diameter was greater than 48 mm, the situation was much more complicated than that when the diameter was less than 36 mm. In this diameter range, the “separation zone” of the boundary layer was located in the mixing chamber. The reattachment point was located in the equal area mixing section, while the “separation point” was located in the mixing chamber. When the primary fluid was ejected through the nozzle, the core of the primary fluid jet expanded continuously and formed a contractile structure in the mixing chamber.

### 4.2. The Effect on the Choking Flow

**Figure 6:**
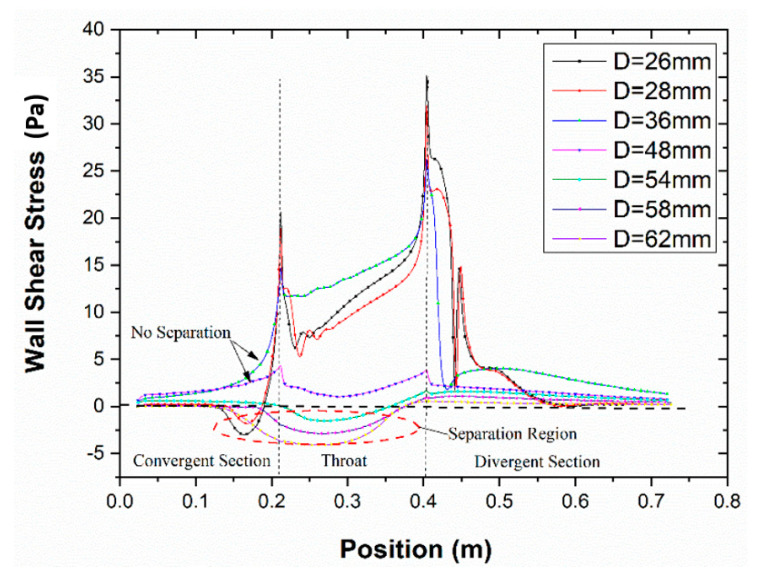
The distribution of wall shear stress via mixer diameter.

The secondary fluid effective area is clearly illustrated due to choking during the fluid flow, as represented in Figure 6. The result highlighted that the secondary fluid effective area grew with the increase of the diameter value. Under the same working conditions, the primary fluid pumped the fluid with the same mass flow rate from the nozzle. When the diameter of the mixing section rose, the relative flow area occupied by the jet core of the primary fluid from the nozzle declined. The flow channel of the ejector vapour enlarged, which led to the extension of its effective area and the increase of the velocity and pressure gradient between the fluid and the walls. The mixing process of the two fluids was weak. Meanwhile, a mass of secondary fluid passed through the congestion position. The boundary layer separated in the mixing section downstream of the mixing chamber.

**Figure 7:**
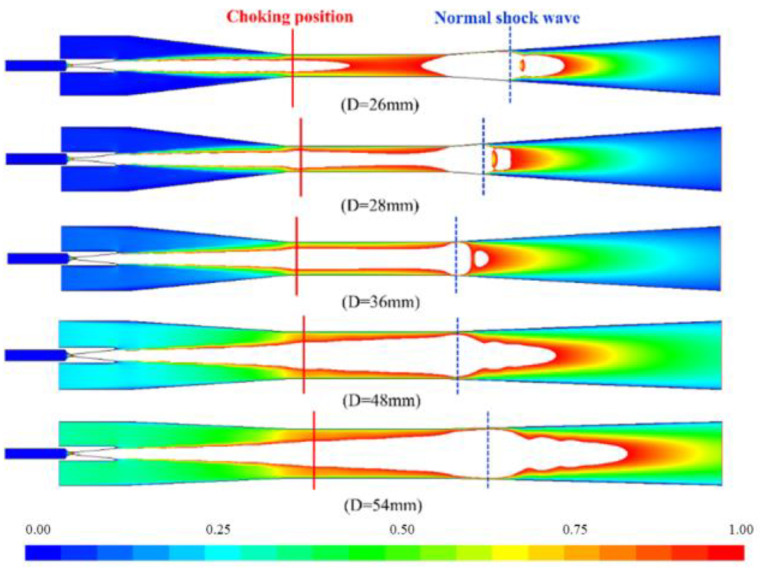
The Mach contours below one fluid under different mixer diameters.

Figure 7 shows the Mach cloud diagram of the ejector at different mixing section diameters. To represent the structure of the primary fluid jet core, the figure only shows the flow field when the Mach value was greater than 1. When the diameter of the mixing section was less than, or equal to, 36 mm, the positive shock wave near the entrance of the diffusion section could avoid flow disturbance. This phenomenon was caused by the variation of back pressure when propagating upstream of the fluid. The back pressure change would not influence the flow channel shape in this case. The location of the choking fluid was still in the mixing section, holding the position of the secondary fluid effective area. At this time, the ejector was in critical working mode. Remarkably, the ejector was operating in double choking fluid mode. This is because the pressure near the wall upstream of the shock wave was uniform and rather small.

In addition, a very thin boundary layer was shaped by the interaction between the mixed fluid and the wall, so no separation occurred between the wall and the fluid. Although the shock wave still existed near the diffusion section entrance, preventing the disturbance produced by back pressure from spreading upstream. With the reduction of effective area size and the existence of a reverse pressure gradient, the mixed fluid flowed in a reverse direction, and the pumping performance decreased rapidly.

The interaction between the secondary and the primary streams was gradually enhanced, resulting in the separation of the mixed fluid from the wall boundary layer. As seen in Figure 7, having a diameter equal to, or greater than, 48 mm, the overall energy of the mixed fluid was weak, and no positive shock wave was generated. During the propagation of the back pressure upstream resulting from the disturbance, the presence of backflow was destroyed, and the ejector was placed in the backflow working mode. As the backflow rose, the choking area disappeared, which, in turn, caused the mixed fluid to fail to mix, thus rendering the ejector ineffective. The analysis of the above results reveals that each ejector has an effective mixing section diameter variation range under the same operating conditions. It works properly when the ejector operates within this effective diameter range.

### 4.3. The Effect on the Entrainment Ratio

**Figure 8:**
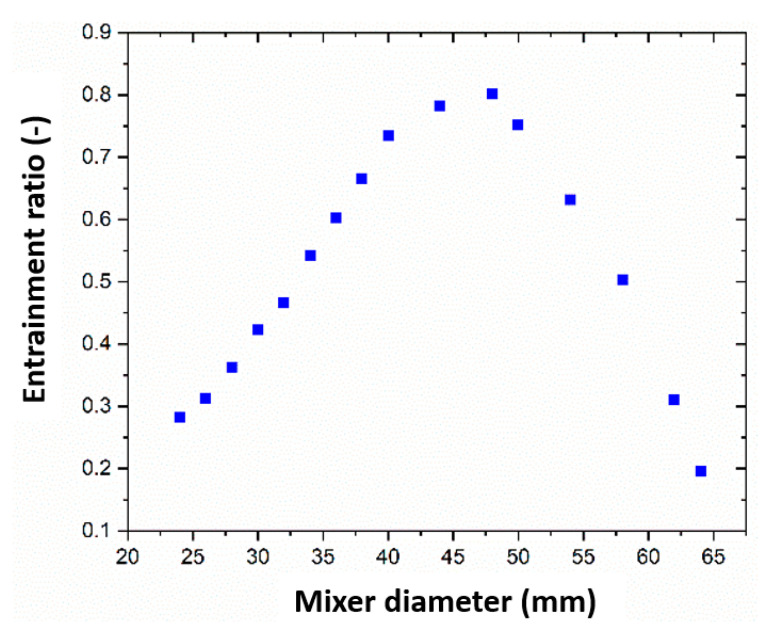
The impact of different mixer diameters on the ejector entrainment ratio.

This section describes two main features. It delivers a compact and precise explanation of the experimental results with clarification and experimental conclusions and assessment of the research. The mixing section diameter is a crucial geometric parameter affecting the efficiency and performance of the steam ejector. Figure 8 illustrates the variation of the ejector entrainment ratio under different diameters at a secondary and primary pressure level of 2330 Pa and 0.34 MPa and back pressure of 3500 Pa. The diameter size varied from 24 mm to 64 mm. The results demonstrated an initial increase in the mass flow rate of the steam ejector then a decrease through the diameter increase. However, the primary mass flow rate was relatively stable, with minor changes which could be considered negligible. The ejector entrainment ratio first increased and then decreased. When the diameter was 48 mm, the mass flow rate and ejector entrainment ratio reached their maximum values. According to the above analysis, when the diameter value of the mixing section was 48 mm, the entrainment ratio reached its maximum, and the ejector efficiency reached its highest under this working condition.

### 4.4. The Effect on the Critical Back Pressure

**Figure 9:**
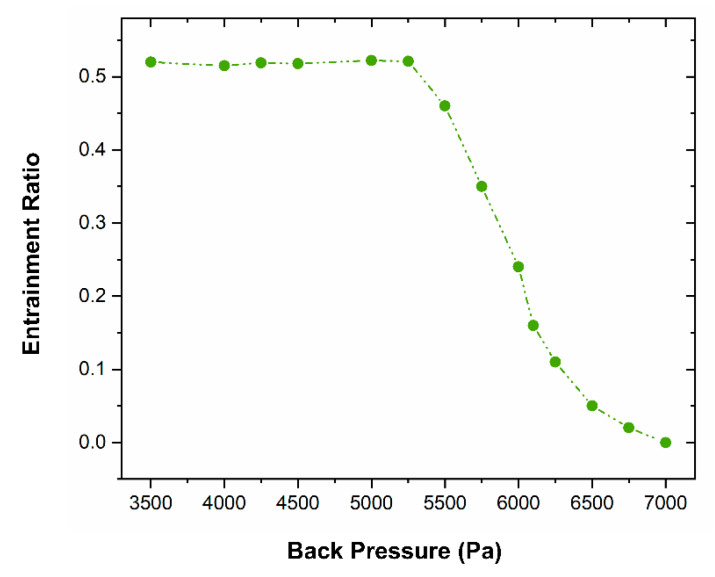
Ejector entrainment ratio and back pressure relationship.

Figure 9 represents the influence of changing the back pressure on the entrainment ratio at primary and secondary pressure levels of 1710 Pa and 0.36 MPa via a mixing section diameter of 48 mm. The result revealed a high ejector entrainment ratio with a maximum value of 0.53 reached at a back pressure of 5300 Pa. However, at lower back pressure, the change in entrainment ratio was slight, and the value remained close to 0.53. On the other hand, having higher back pressure led the entrainment ratio value to drop sharply until the back pressure reached 7000 Pa. This indicated that the back pressure of 5300 Pa represented the ejector critical back pressure value under this operating condition. Once the back pressure was below 5300 Pa, the ejector was in a normal working state.

When the ejector back pressure exceeded 5300 Pa, the ejector’s pumping performance decreased until it failed (i.e., when the entrainment ratio was 0). Therefore, in the actual design process of the ejector, not only the stability of the exhaust efficiency but also the limited exhaust capacity should be taken into account. The front pump of the experimental system provides a low enough front pressure, which provides conditions for the improvement of the exhaust efficiency of the steam ejector.

## 5. Conclusions

In this study, CFD simulation was performed to investigate the influence of the mixing section diameter on the ejector performance. In addition, the fluid characteristics and the ejector pumping performance were examined at different mixing diameters. The reasons for ejector failure were evaluated, and a criterion for judging the critical back pressure was recommended. The key conclusions of this research can be stated as follows:

1) The research results show that the high primary pressure of the ejector reduces the secondary fluid flow area. The entrainment ratio of the ejector decreases accordingly.
2) Under certain operating conditions, expanding the diameter of the mixing section, such as the diffuser, can improve the pumping performance of the ejector and reduce its ultimate exhaust capacity. When the diameter increases from 24 mm to 28 mm, the entrainment ratio of the steam ejector improves by 89.29%, but the critical back pressure of the steam ejector drops by 21.43%.
3) When the diameter of the mixing section is less than 48 mm, the entrainment ratio increases gradually with the diameter increase. When the diameter of the mixing section is larger than 48 mm, the ejector entrainment ratio decreases with the growth of the diameter. When the diameter is 48 mm, the entrainment ratio reaches the maximum value of 0.81.
4) Under certain working conditions and diameters, the entrainment ratio remains unchanged. Currently, the back pressure value is the critical back pressure value. Beyond this value, the entrainment ratio plummets until the ejector becomes a failure entirely.
5) For a specific steam ejector fire extinguishing system, the optimal structure with the highest exhaust efficiency can be found by optimizing the crucial geometric parameters of the ejector on the premise of considering the exhaust efficiency and the limited exhaust capacity.

## Figures and Tables

**Table 1:** Steam ejector main geometry parameters.

Parameter	Value and Unit
Primary nozzle inlet diameter	12 mm
Primary nozzle outlet diameter	11 mm
Primary nozzle throat diameter	2.5 mm
Nozzle expanded angle	10°
Nozzle exit position	10 mm
Mixing chamber inlet diameter	70 mm
throat diameter	28 mm
Mixing chamber length	122.2 mm
Throat length	90 mm
Subsonic diffuser length	210 mm

## Data Availability

The research data supporting this publication are provided within this paper.
